# A question of TiME: how microenvironmental interactions shape response to immunotherapy in CLL and Richter Transformation

**DOI:** 10.3389/fimmu.2025.1592574

**Published:** 2025-05-29

**Authors:** Alexander F. vom Stein, Phuong-Hien Nguyen, Elisa ten Hacken

**Affiliations:** ^1^ Faculty of Medicine and University Hospital Cologne, Department I of Internal Medicine, Center for Integrated Oncology Aachen Bonn Cologne Duesseldorf, University of Cologne, Cologne, Germany; ^2^ Center for Molecular Medicine Cologne, University of Cologne, Cologne, Germany; ^3^ Center of Excellence on Cellular Stress Responses in Aging-Associated Diseases (CECAD), University of Cologne, Cologne, Germany; ^4^ Department of Medicine, Division of Hematology and Oncology, Weill Cornell Medicine, New York City, NY, United States

**Keywords:** microenvironment, chronic lymphocytic leukemia, Richter Transformation, immunotherapy, mouse models, preclinical studies, clinical trials

## Abstract

Immunotherapy has revolutionized the treatment landscape for many cancers, including some B- cell lymphomas. Immune checkpoint blockade, CAR-T cells and bispecific antibodies have shown promise for the treatment of Richter Transformation (RT) but have displayed reduced activity in chronic lymphocytic leukemia (CLL). These observations suggest that, besides the intrinsic differences between CLL cells and transformed RT cells, there are also marked differences in tumor immune microenvironmental (TiME) composition and tumor-immune cell interactions between these two entities, which remain to be fully characterized. In this perspective, we highlight recent studies describing the TiME in CLL and RT, utilizing both patient-derived tissues and novel mouse models. We then provide a brief overview of current clinical trials employing immunotherapy in CLL and RT and offer a perspective on current challenges and future research efforts in the field.

## 1 Introduction

Chronic lymphocytic leukemia (CLL) remains the most common adult leukemia in Western countries, characterized by substantial variability in patient characteristics and clinical outcomes (1). Treatment of CLL has witnessed remarkable success with targeted therapies, including the use of BTK and BCL2 inhibitors (1). However, a subset of patients develop resistance to these treatments, resulting in poor clinical outcomes without established therapeutic options (2). Another challenge in the clinical management of CLL is the emergence of Richter Transformation (RT), an aggressive B-cell lymphoma occurring in up to 10% of patients, which is associated with dismal clinical outcomes (~12 months median survival) and displays refractoriness to most existing therapies (3).

Despite significant progress in immunotherapies thanks to the introduction of immune checkpoint inhibition, CAR-T cells, and bispecific antibodies (BsAbs), clinical responses to these new agents in CLL have stayed behind those observed in other B-cell malignancies. Importantly, some of these approaches have shown more promise in RT, implying that the tumor immune microenvironment (TiME) in CLL and RT is fundamentally distinct, with the RT-TiME enabling enhanced response to immunotherapy. To better understand these biological and therapeutic disparities, refined model systems and a deeper exploration of the TiME in both preclinical and clinical settings are necessary. Here, we discuss emerging transgenic mouse models for these entities, summarize the current landscape of targeted immunotherapies in CLL and RT, and offer a perspective on how insights from these systems can inform future clinical trials.

## 2 Advances in modeling CLL and RT *in vivo*

### 2.1 Immuno-competent mouse models of CLL and RT

**Figure 1:**
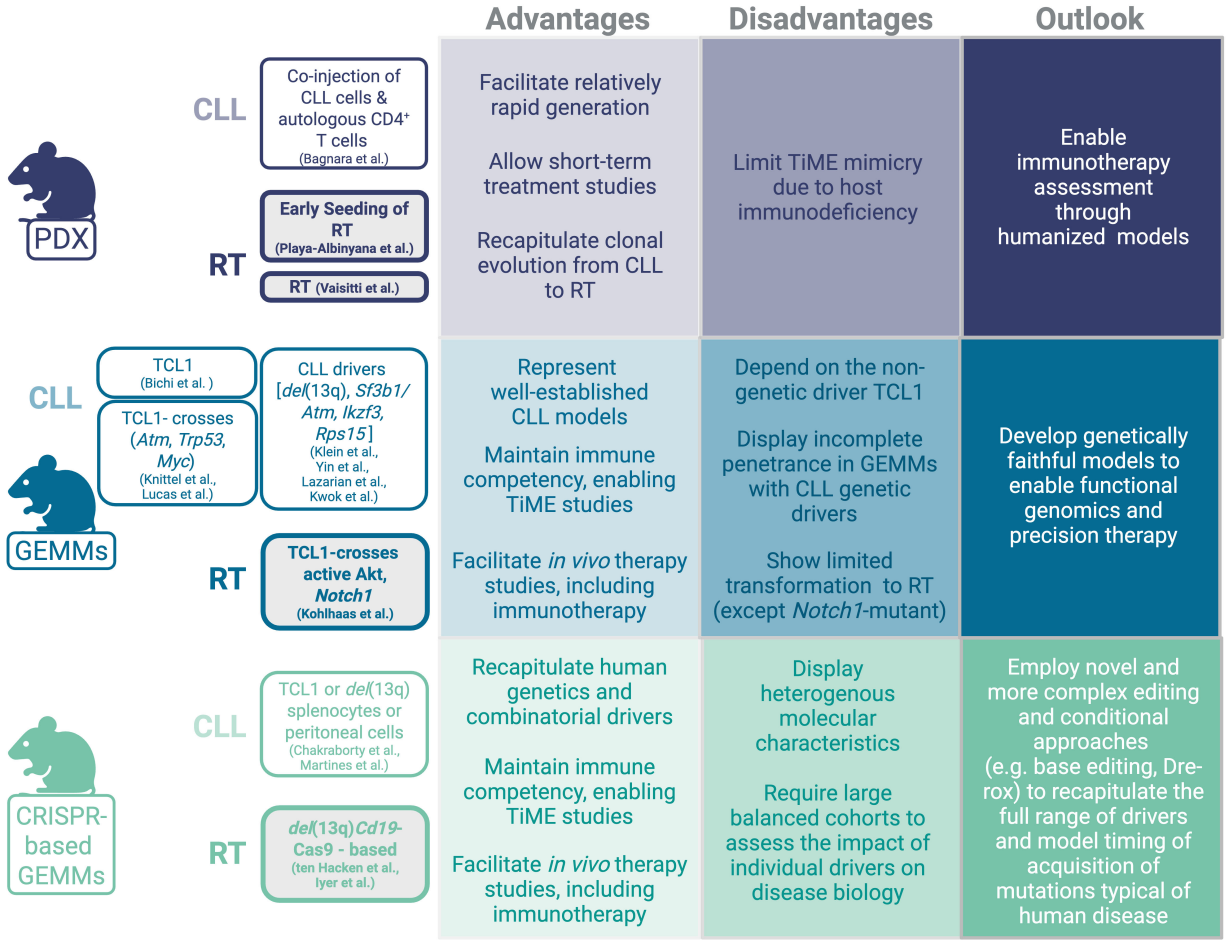
Patient-derived xenograft and genetically-engineered models for CLL and RT.

Mouse models of CLL have been extensively developed and studied, yielding major advancements in our understanding of CLL biology (**Figure 1**). A variety of models capturing the spectrum of disease progression from early-stage monoclonal B-cell lymphocytosis (MBL) to advanced, aggressive disease have provided critical insights into disease pathogenesis and preclinical therapeutic response. Among them, the Eµ-TCL1 transgenic mouse remains the most commonly employed, owing to its high disease penetrance (100%) and well-characterized disease characteristics, which are recognized to recapitulate an aggressive variant of CLL-like disease (4). Conditional knock-in/out strategies have been used to model CLL genetic drivers including *del*(13q) *(* 5), *Sf3b1/Atm* co-mutation (6), *Ikzf3 (* 7) and *Rps15* (8), all of which lead to incomplete disease penetrance but faithful disease characteristics to indolent CLL-like disease (as previously reviewed) (9).

Novel immuno-competent mouse models that recapitulate CLL transformation into RT are now also available. Some of the initial studies have relied on intercrosses based on the Eµ-TCL1 background and provided functional insight into the relevance of selected genetic drivers and/or signaling pathways in RT, including loss or *Trp53* or *Atm* (10), MYC overexpression (11), and aberrant expression of the protein arginine methyltransferase (PRMT5) (12). However, in these early models, RT was observed only in less than 20% of mice lacking *Trp53*, and none in mice lacking *Atm*, potentially due to the small size of the analyzed cohorts (10), while MYC overexpression resulted in a clonally-unrelated lymphoma co-developing alongside CLL rather than arising through a clonally-related transformation process (11). More recently, B-cell specific activation of *Akt* or *Notch1* in the Eµ-TCL1 transgenic background resulted in 100% RT and significantly shortened survival compared to Eµ-TCL1 mice, thus showing the highest reported transformation rate among CLL mouse models (13). Notably, NOTCH signaling hyper-activation mediated by AKT constitutive activation on the Eµ-TCL1 background was associated with increased interaction of the transforming clone with CD4^+^ T-cells overexpressing the NOTCH ligand DLL1, thus providing a functional link between presence of selected molecular changes and their impact on tumor microenvironmental reprogramming.

Several studies have now implemented the CRISPR/Cas9 technology to model complex co-occurring alterations typical of RT via either stem-cell engineering of *del*(13q)-*Cd19*Cas9 donor mice [i.e., animals expressing Cas9-GFP in a B-cell restricted fashion together with the leukemogenic *del*(13q)- background] (14, 15) or of splenocytes or peritoneal cells derived from either Eµ-TCL1 or *del*(13q) mice (16, 17). Modeling combinatorial molecular drivers directly in stem cells or B-cells allowed faithful recapitulation of the molecular events leading to clonally-related RT arising from antecedent indolent CLL. Collectively, these models provided important knowledge on the functional role of recurrent genetic drivers of RT discovered through large-scale next generation sequencing studies (18, 19), including the MYC signaling regulator *Mga*, recently demonstrated to contribute to aberrant mitochondrial oxidative phosphorylation and glycerolipid metabolism via upregulation of the MYC target *Nme* (14, 20). Recent studies have also demonstrated the relevance of cooperative events in transformation biology, including co-occurrence of mutation in the ribosomal protein *Rps15* and in the *Trp53* tumor suppressor which jointly alter DNA damage response pathways (8), and the presence of autoreactive (i.e. ‘stereotyped’) B-cell receptors in models carrying concomitant alterations in *Trp53* and the cell cycle regulators *Cdkn2a/b* (21), implying chronic auto-antigenic stimulation as a predisposing factor for transformation. The notion that B-cell receptor (BCR) stimulation may be implicated in transformation is further supported by the evidence that loss of the anergy regulator NFAT2 led to transformation into RT *in vivo* (22). Importantly, all these newly developed mouse models are fully immuno-competent and transplantable into syngeneic recipients, thus providing a valuable platform amenable to short-term (1-2 months) treatment studies, including of novel immunotherapies.

### 2.2 Patient-derived xenograft models of CLL and RT: recent advances and current challenges

In contrast to the wide range of transgenic mouse models, developing patient-derived xenograft (PDX) models for CLL and RT has proven to be significantly more challenging (**Figure 1**). CLL-PDX models could be established when neoplastic CLL cells were co-injected with autologous pre-activated CD4^+^ T-cells in NSG mice (23). CD4^+^ T-cell co-injection supported the engraftment of CLL cells in the perivascular area of spleens and highlighted the functional contribution of CD4^+^ T-cells to CLL progression. Although these PDX models successfully recapitulated the activation of NF-ĸB and BCR signaling in malignant B-cells, mimicking what is typically observed in the CLL lymph node microenvironment, the incomplete homology of murine and human signaling receptors and cytokines/chemokines limited the faithful recapitulation of key microenvironmental interactions. Although CLL engraftment in these PDX models could not persist long-term, these models have proven to be useful to investigate efficacy of targeted therapies, including BTK inhibitors (24).

The relatively rare occurrence of RT (~2-10% CLL patients) and the general difficulties in tissue procurement have thus far limited the generation of stable cell lines for *in vitro* functional analyses –with the sole exception of the U-RT1 cell line – (25) and of PDX models. Nonetheless, some RT-PDX models were thus far generated and have allowed the assessment of novel therapeutic modalities, including the combination of PI3K inhibition via duvelisib with BCL2 inhibition by venetoclax (26), anti-ROR1 monoclonal antibodies (27), anti-CD37 immunotoxins (28), or BET-PROTACs alone or combined with the BTK inhibitor ibrutinib or the BCL2 inhibitor venetoclax (29). More recently, PDX models were used to validate the existence of an ‘early seeding’ process in RT, defined as presence of subclonal ‘RT-like’ populations already present in CLL samples up to ~20 years prior to clinical and histological diagnosis of RT in patients (19). By engraftment of cells at the CLL stage into NSG recipients, Playa-Albinyana et al. observed transformation into clonally-related RT *in vivo*, further demonstrating clonal-relatedness of the two malignancies and providing proof-of-principle for the transforming potential of RT seeds (30). While PDX models represent valuable tools for the pre-clinical evaluation of therapeutics, particularly those targeting human antigens (e.g., anti-CD37 immunotoxins), the lack of functional T-, B- and NK-cells characterizing the NSG strain limits the ability to interrogate immune-related changes underlying response and resistance to immunotherapy. It is tempting to speculate that the TiME might contribute to the suppression of early RT seeds, thus the lack of immune surveillance after transplantation into NSG mice might facilitate this outgrowth process. To this end, novel humanized mouse models generated via engraftment of human stem cells and subsequent repopulation of the recipient mouse with a functional human immune system are underway (31) and will allow the evaluation of efficacy of novel immunotherapeutic strategies on both tumor-intrinsic and tumor-extrinsic pathways at greater depth.

## 3 The distinct TiME of RT in comparison to CLL

**Figure 2:**
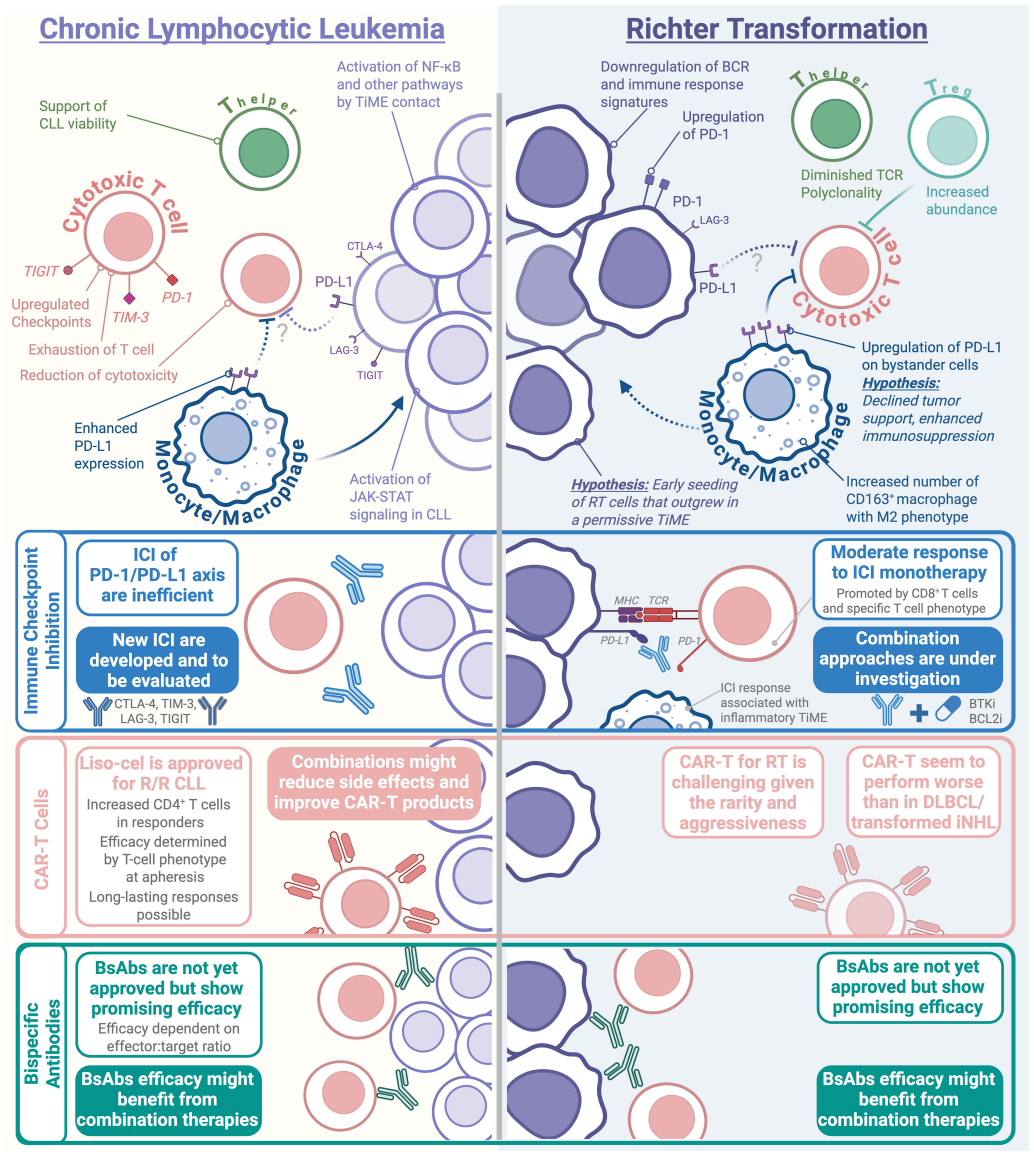
The human CLL and RT TiME and immunotherapeutic strategies.

The TiME has long been understood to play a crucial role in CLL initiation and progression (**Figure 2**). Bi-directional interactions with different cell types in the tissue microenvironment, in particular macrophages and stromal cells, promote CLL survival and resistance to therapy by activation of BCR, NF-ĸB and Toll-like receptor (TLR) signaling, as well as upregulation of antiapoptotic proteins in leukemic cells. In addition, CD4^+^ T-cells significantly support CLL-cell proliferation and disease progression, while the cytotoxic capacity of CD8^+^ T-cells and NK-cells is limited, thus allowing leukemic cells to evade killing (32, 33). T-cell exhaustion with upregulation of immune checkpoint molecules such as PD-1, TIGIT or TIM-3 on T-cells (34, 35), TiME-interaction-induced PD-L1 and CTLA-4 expression on CLL cells (36), and proliferative signaling (37) contribute substantially to immune evasion. Abundance of precursor exhausted CD8^+^ T cells is a characteristic feature of the CLL lymph node TiME, and upregulation of Galectin 9 (the ligand for TIM-3) on CLL tumors is associated with inferior CLL patient survival (38). Interestingly, immunohistochemical (IHC) studies detect only minimal PD-L1 expression on the malignant cells, instead PD-L1 is predominantly expressed on non-malignant bystander cells, such as macrophages (39, 40). This observation is even more pronounced in RT than in CLL samples (41) and indicates further contribution of other TiME cells to immune suppression in both entities. Recent data demonstrates that RT preserves a core CLL-specific gene expression profile, which includes upregulated genes involved in the BCR signaling and downregulated genes related to immune response, TP53 signaling, and the JAK-STAT pathway (42). Kohlhas et al. have recently shown that macrophage interactions can activate the JAK-STAT pathway in CLL cells (43), highlighting the critical role of macrophages in enhancing CLL viability via inflammatory signaling.

Key insights into TiME composition of RT have primarily been gathered via IHC analysis of lymph node tissues, including increased presence of regulatory T-cells and CD163^+^ tumor-associated macrophages (41), together with enhanced PD-1 staining on clonally-related malignant B-cells. PD-1 expression on malignant B-cells appears to be a unique characteristic of human (and murine) RT, that is generally not observed in CLL or *de novo* DLBCL (44, 45). Diminished T-cell receptor (TCR) polyclonality has also been observed in RT compared to CLL (41), which suggests presence of common antigenic determinants underlying evolution from antecedent CLL, that –together with BCR polyreactivity– further supports the relevance of antigenic drive in transformation biology. More recently, single-cell RNA sequencing studies of bone marrow from RT patients showing differential response to checkpoint blockade therapy allowed the identification of a population of CD8^+^ effector/effector memory T-cells, which were marked by the transcription factor *ZNF683*, and which displayed preserved cytotoxicity and intermediate exhaustion, in association with response to therapy. Similarly, baseline peripheral T-cells in responding patients overexpress *ZNF683* and PD-1, whereas non-responding patients demonstrated upregulation of NK-/T-cell-related genes (46). In separate studies, RT patients responding to checkpoint inhibition show increased abundance of circulating CD8^+^ T-cells with reduced exhaustion markers and an interferon-gamma (IFN-γ) signature (47), which is consistent with *in vitro* observations of increased IFN-γ secretion by activated T-cells upon PD-1/PD-L1 blockade (34). A recent study cross-compared TiME characteristics of murine RT splenocytes and human RT lymph nodes identifying abundant (and shared) presence of pro-inflammatory CXCL9^+^ tumor-associated macrophages and CD8^+^ PD-1^+^ polyfunctional T-cells in patient and murine samples showing favorable response to checkpoint blockade therapy with anti-PD-1 (48). In line with the known relevance of monocyte/macrophages in mediating CLL progression *in vivo* in mouse models (49), TLR inhibition via IRAK4 blocking reduces macrophages and delays RT cell engraftment *in vivo* (17). Despite the increased infiltration of M2-skewed CD163^+^ macrophages in RT compared to CLL (41), the role of macrophages in RT seems less important for direct tumor cell support in patient samples. Although the functional significance of these macrophages in RT patients is not yet well-defined, their high PD-L1 expression (41) suggests a stronger contribution to immune suppression rather than direct tumor survival support. A thorough phenotypic and functional analysis of RT-associated macrophages would be invaluable to clarify their precise contributions to RT.

## 4 Clinical insights into immunotherapeutic approaches in CLL and RT

Restoring immune surveillance by rewiring the immune-suppressive TiME is a major goal for treating CLL and RT. Since CLL cells strongly promote an exhausted T-cell phenotype, therapy with BCL2 and BTK inhibitors (BCL2i/BTKi) that efficiently reduce leukemic burden generally help to restore T-cell functionality (50–52). The pronounced beneficial effects of BTKi and other kinase inhibitors on the T-cell compartment —partially mediated by off-target effects on T-cell specific kinases— make them attractive combination partners to enhance immunotherapy efficacy (2), as currently tested in clinical trials for both CLL and RT (**Figure 2**).

### 4.1 Immune checkpoint inhibition

The biological differences in the TiME are particularly reflected in different clinical responses to immune checkpoint inhibitors (ICIs). Single-agent pembrolizumab showed no efficacy in relapsed CLL patients (40), but achieved modest responses with limited durability in RT patients (objective response rate [ORR] ~10–44%) (40, 53). Notably, in cases of concurrent CLL and RT, ICIs selectively improved the RT phase while sparing the CLL phase, emphasizing differences in ICI susceptibility of these two entities, even when they co-exist within the same patient (40). Evaluating a BTKi plus ICI combination (Ibrutinib plus Nivolumab) in high-risk CLL showed comparable responses to BTKi monotherapy, corroborating the inefficiency of ICI in CLL (54). Subsequent combination therapies have demonstrated greater promise in RT. Trials combining ICIs with BTKi or PI3K inhibitors have yielded response rates of 42–65%, with longer progression-free survival compared to ICI or kinase inhibitor monotherapy (47, 54–56). The mechanisms by which BTKi enhance the efficacy of ICI in RT remains unclear and is somewhat surprising. This is particularly notable given that: (i) BTKi monotherapy demonstrated moderate activity in RT (ORR 40% for acalabrutinib and 50% for pirtobrutinib) but responses were only short-lived with median durations of 6-8 months (57, 58), (ii) preclinical studies have indicated reduced dependence of RT cells on BCR signaling (19), and (iii) most RT patients had prior exposure to BTKi as part of CLL treatment (e.g., 66% in the RT1 trial, 25% in the MOLTO trial), although the relevance of BTKi resistance mutations for RT transformation compared to those occurring in the CLL phase is incompletely understood (59). Biomarker analyses from trials of BTKi-ICI combinations revealed only a weak correlation between baseline PD-1/PD-L1 expression and clinical responses (39, 40, 54). Additional combinatorial strategies are being explored, including the potential of triple combinations. The MOLTO trial demonstrated the efficiency of combining BCL2i (Venetoclax) with ICI (anti-PD-L1 antibody Atezolizumab) plus a CD20 Antibody (Obinutuzumab), resulting in similar ORR and lasting responses as BTKi plus ICI (39). Consequently, ongoing trials are investigating triple combinations involving ICI, BTKi and BCL2i (NCT04271956 and NCT05388006).

Besides the PD-1/PD-L1 axis, other immune checkpoints such as LAG-3, TIGIT and CTLA-4 are overexpressed on CLL cells, contribute to immune evasion and are potential targets for immunotherapy. CTLA-4 expression on leukemic cells is regulated by microenvironmental interactions (36); contact with activated CD4^+^ T cells upregulates, whereas contact with stroma cells and the lymph node TiME reduces its expression. Inhibition of CTLA-4 reduces tumor burden in a murine model and augmented cytotoxicity of bispecific antibody treatment *in vitro* (36, 60, 61). Similarly, LAG-3 inhibition in combination with PD-1 inhibition reduces tumor burden *in vivo* (62). Targeting Galectin 9, the ligand for TIM-3, has also shown promise in preclinical studies in the Eμ-TCL1 mouse model (38). To our knowledge, no clinical trial has tested corresponding ICI in CLL or RT so far, but they represent promising therapeutic targets in upcoming combination therapies.

### 4.2 Chimeric antigen receptor T-cells

Overcoming immune evasion by CAR-T-cells, has been extensively investigated in CLL and RT. Based on the pivotal phase 1-2 TRANSCEND CLL 004 trial (63), lisocabtagene maraleucel (liso-cel) was approved for treatment of relapsed CLL after prior BTKi and BCL2i therapy (double-exposed) by the FDA in March 2024. In this trial, 43% of double-exposed CLL patients responded to CAR-T therapy, 18% achieving a complete response (CR), with a median duration of response of 35 months. Although response rates for CLL are much lower compared to other B-cell lymphomas, responses in CLL can be very lasting. Most patients, who reach one year of progression-free survival (PFS) after infusion, remain progression-free for more than 5 years without further treatment (64). Characterization of such long-lasting responders identified an active, proliferative CD4^+^ CAR-T population with cytotoxic characteristics (65). Similarly, the infusion product in responding patients had significantly more CD4^+^ cells and less effector/memory-like CD8^+^ T-cells than in non-responding patients in a trial using a third generation academic CAR-T construct (66), indicating a role for cytotoxic CD4^+^ T-cells for lasting leukemia control. In addition, characterization of infusion products and immune composition at time of apheresis in responding patients shows increase of early-memory T-cell phenotypes, increased IL6/STAT3-signature, reduced T-cell exhaustion and effector differentiation, as well as abundance of a CD45RO^-^ CD27^+^ CD8^+^ T-cell population (67). This demonstrates that the T-cell characteristics at time of apheresis are highly relevant for successful CAR-T cell therapy, and the generally strongly exhausted CLL T-cell compartment might underlie the low response rates for CAR-T in CLL compared to other B-cell lymphomas. In addition, a persisting residual lymphadenopathy upon CAR-T treatment, even in patients that achieve undetectable minimal residual disease (uMRD) in peripheral blood or bone marrow, indicates a role for an immune-suppressive TiME in limiting efficacy of CAR-T cells (63, 68). Macrophages, fibroblasts and other stromal cells are important components of the CLL microenvironment and have been associated with suppression of CAR-T cells in other entities (69, 70).To overcome these challenges, combination of CAR-T cells with BTKi is clinically feasible and safe (68), and data indicates a trend towards increased response rates (71, 72), and facilitated CAR-T-cell manufacturing after Ibrutinib pre-treatment (73). Moreover, CAR-T therapy in CLL is hampered by frequent side effects including cytokine release syndrome (CRS) and neurotoxicity and BTKi treatment might lower CRS rates (71, 72), although randomized trials are still needed to clearly confirm these effect.

Given the rarity of RT, little insight on CAR-T cell treatment has been collected in prospective trials and one prospective trial on the use of Brexucabtagene autoleucel (brexu-cel) in relapsed/refractory RT was stopped before full recruitment. In a large retrospective, multicenter analysis with different CAR-T cell products approved for DLBCL, treatment of RT demonstrated an ORR of 63% with 46% achieving CR. While median PFS was short (4.7 months), patients achieving a CR (46%) had a median duration of response of 27.6 months, indicating that CAR-T can induce comparatively longer responses in RT patients (74). However, compared to *de novo* DLBCL or transformed indolent non-Hodgkin lymphoma, RT shows reduced response rates and is a significant negative prognostic factor in multivariate analyses (75). Thus, current trials are aiming to improve response rates by combination of CAR-T cells with BTKi (NCT05873712), as well as BTKi and ICI (NCT05672173).

### 4.3 Bispecific antibodies

Emerging clinical trials in both CLL and RT are exploring the potential of BsAbs, which target CD3 on T-cells and CD19 or CD20 on B-cells thereby inducing T-cell-mediated cytotoxicity. Preclinical studies show that CD19/CD3 and CD20/CD3 BsAbs effectively induce CLL lysis *in vitro* and in PDX models, with their efficacy linked to effector-to-target ratios (76, 77). Clinically, the anti-CD20/CD3 BsAb Epcoritamab has shown promising activity in refractory, high-risk CLL patients, with an ORR of 61%, albeit with high CRS rates (78). Other BsAbs, such as the anti-CD20/CD3 Mosunetuzumab, are currently under evaluation (NCT05091424). BTKi pre-treatment can enhance BsAb-mediated cytotoxicity, demonstrating that interfering with CLL-associated immuno-suppression by combination approaches is a promising future treatment strategy (50, 76). BsAbs also synergize with CAR-T cells in murine CLL models, achieving complete leukemia ablation and prolonged survival compared to monotherapy (79), although the risk of exacerbated CRS or immune effector cell-associated neurotoxicity syndrome (ICANS) requires careful clinical validation. BsAbs are also effective in RT, demonstrating ORRs of up to 63% with the anti-CD20/CD3 Glofitamab in pre-treated patients (80–82). While early relapse is frequent in non-CR patients, patients achieving CR often sustain responses for over 20 months, potentially reflecting restored immunosurveillance. However, CRS rates remain a concern, reaching 80% with Epcoritamab (82). The anti-CD19/anti-CD3 bispecific T-cell engager Blinatumomab combined with R-CHOP has been shown to improve response depth in patients who did not initially achieve CR (80), highlighting a potential avenue for a new combination approach. Ongoing trials are investigating novel strategies, such as BsAbs with ICIs (NCT06043674) or BTKi (NCT06735664), to further improve efficacy.

## 5 Perspective

### 5.1 Deep multi-omic profiling of patient samples

A critical next step in advancing immunotherapy for CLL and RT lies in the comprehensive characterization of TiME characteristics in RT patients in direct comparison to CLL cohorts. The rapid development of high-throughput single-cell and spatial analytics has opened new possibilities for in-depth profiling of different immune cell subsets, offering unprecedented resolution in mapping cellular interactions and signaling networks. These analyses will be crucial for uncovering the fundamental differences in TiME between CLL and RT, ultimately guiding more precise TiME-focused therapeutic strategies. Genotype-aware multi-omics (e.g. Genotyping-of-Transcriptomes, Genotyping-of-Targeted *loci* with single-cell Chromatin Accessibility) (83, 84) will further allow to dissect the function of individual (or multiplexed) driver mutations when in complex admixtures, such as those occurring in CLL specimens evolving into RT. To this end, accurate biobanking of longitudinal samples and/or of lymph node material from CLL and paired RT will be essential, as RT specimens are currently mostly collected as paraffin-embedded tissue, while most multi-omic strategies require viable single-cell suspensions. Nodal single-cell suspensions could also be valuable to generate 3D cell culture models or organoids, a rapid tool to screen personalized therapeutics. These systems are just emerging for CLL (85, 86), as they have been notoriously challenged by the poor *ex vivo* viability of primary CLL patient-derived material.

### 5.2 Refinement of preclinical *in vivo* and *ex vivo* models

In parallel to the patient-focused studies, these comprehensive TiME analyses will also benefit substantially from the recently generated RT mouse models that faithfully recapitulate the transformation process, which offer a more controlled sampling setting along the longitudinal evolution of CLL into RT. Novel genetically-engineered mouse models also provide a fundamental tool to dissect the mechanistic relevance of genetic drivers in RT, which is still largely lacking. Incorporating novel CRISPR-based editing strategies such as cytosine base editing (87) will further allow a more comprehensive modeling of disease drivers, which is now limited to loss-of-function mutations achieved via conventional Cas9-based methods. Addition of inducible engineering strategies (e.g. Dre-rox) (88) would further facilitate the modeling of timing of acquisition of driver events typical of human disease. The development of well-annotated PDX models will further be advantageous to identify novel targets and personalized treatment strategies, enabling the rapid assessment of drug sensitivities and resistance patterns. Humanized mouse models, particularly those that enable reconstitution of the human myeloid compartment (89), represent a central tool for preclinical evaluation not only of T-cell-based immunotherapies but also of macrophage-reprogramming strategies. A key advantage of humanized mouse models is the faithful recapitulation of the human immune system, which can guide preclinical assessment of novel immunotherapies in a personalized manner and anticipate possible risks/side effects.

As patients with double-refractory CLL and RT have limited therapeutic options and dismal outcomes, immunotherapies hold promise for addressing this critical unmet need. Significant progress is being made to establish such therapies, yet a key challenge remains the relatively poor response of CLL. Preclinical studies will be essential to pave the way and improve these outcomes, and these can benefit from either faithful GEMMs or 3D/organoid models. Advanced culture systems for CLL have progressed from co-cultures with stromal- or nurse-like cells (43, 90, 91) to more sophisticated 3D multicellular spheroid assemblies that better recapitulate lymph node architecture and cellular interactions (86, 92). These spheroids incorporate multiple adaptive immune cells with leukemic cells and can provide a more physiologically relevant platform to study drug responses. The integration of 3D bioprinting, which supplements a close-to-native extracellular matrix, further offers significant advantages to faithfully recreating the complex structure of lymphoid tissues (92). Such culture systems for RT remain underdeveloped due to the scarcity of primary material. Adapting these advanced CLL culture models and employing 3D DLBCL spheroid models (69) will be valuable for dissecting RT-TiME interactions and evaluating therapeutic approaches preclinically.

### 5.3 Advanced diagnostic tools to guide novel combination therapies

Companion diagnostic tools derived from preclinical analyses, such as expression of *ZNF683* in blood PBMCs (46), presence of IFN-γ signatures in the T-cell compartment (47), pro-inflammatory TiME signatures and PD-L1 expression on tumor cells or macrophages (48), could be incorporated in translational screening platforms to identify patients with a higher likelihood of exhibiting a favorable response to immunotherapy. Applying such pre-clinically defined diagnostic approaches along novel clinical trials, combined with thorough characterization of the TiME adaptations under treatment, holds promise not only for patient-specific prognostication of response to novel immunotherapies but also to identify novel combination treatments that promote induction of these TiME signatures. Such combination regimens, for example those integrating the use of kinase inhibitors, show potential to overcome resistance mechanisms mediated by the TiME and have already demonstrated to enhance immunotherapeutic efficacy in RT. Notably, combinations of novel BTK inhibitors with BsAbs or CAR-T cells —all of which exhibit single-agent activity in refractory disease— are now being planned in CLL. Moreover, innovative therapies must be increasingly tailored to specific targets identified through preclinical research. Next generation ICI intercepting CTLA-4, TIGIT, LAG-3 or TIM-3, are being developed and demonstrate preclinical potential in CLL and can be further tested in novel murine models and clinical trials. Another emerging concept involves combining multiple immunotherapeutic agents to augment anti-tumor responses. For instance, the combination of BsAbs with CAR-T cells, shown to be highly effective in preclinical studies (79), or integrating these modalities with ICIs, may further enhance immunotherapeutic potency as preclinically demonstrated. However, these novel approaches warrant careful safety monitoring given the risk of exacerbated CRS and ICANS. Here, novel immune-competent GEMMs and humanized murine models can further contribute to pre-clinical assessment of safety of combination strategies. Robust translational research must accompany ongoing clinical trials. This synergy between bench and bedside will provide real-time insights into the immunological shifts occurring in patients and foster the development of rational combination regimens designed to maximize efficacy and minimize toxicity.

## Data Availability

The original contributions presented in the study are included in the article/supplementary material. Further inquiries can be directed to the corresponding author.
